# Linking coral fluorescence phenotypes to thermal bleaching in the reef-building *Galaxea fascicularis* from the northern South China Sea

**DOI:** 10.1007/s42995-023-00190-1

**Published:** 2023-10-18

**Authors:** Sanqiang Gong, Jiayuan Liang, Gang Li, Lijia Xu, Yehui Tan, Xinqing Zheng, Xuejie Jin, Kefu Yu, Xiaomin Xia

**Affiliations:** 1 Key Laboratory of Tropical Marine Bio-Resources and Ecology & Guangdong Provincial Key Laboratory of Applied Marine Biology, South China Sea Institute of Oceanology, Chinese Academy of Sciences, Guangzhou, 510301 China; 2 Southern Marine Science and Engineering Guangdong Laboratory (Guangzhou), Guangzhou, 510301 China; 3 Coral Reef Research Center of China, Guangxi University, Nanning, 53004 China; 4 South China Institute of Environmental Sciences, The Ministry of Ecology and Environment of PRC, Guangzhou, 510530 China; 5 Third Institute of Oceanography, Ministry of Natural Resources, Xiamen, 361005 China

**Keywords:** Fluorescence phenotype, Thermal bleaching, Microbiome, *Galaxea fascicularis*, Ocean warming

## Abstract

Coral fluorescence phenotypes have been suggested as an adaptation to a broad range of environmental conditions, yet the mechanisms linking thermal bleaching tolerance in reef-building coral populations, associated with fluorescence phenotypes due to GFP-like proteins, remains unclear. In this study, the relationship between the thermal sensitivity and phenotypic plasticity of corals was investigated using two phenotypes of *Galaxea fascicularis,* green and brown. The results reveal that brown *G. fascicularis* was more susceptible to bleaching than green *G. fascicularis* when exposed to a higher growth temperature of 32 °C. Both phenotypes of *G. fascicularis* were associated with the thermotolerant Symbiodiniaceae symbiont, *Durusdinium trenchii*. However, the brown *G. fascicularis* showed a significant decrease in Symbiodiniaceae cell density and a significant increase in pathogenic bacteria abundance when the growth temperature was raised from 29 to 32 °C. The physiological traits and transcriptomic profiles of Symbiodiniaceae were not notably affected, but there were differences in the transcriptional levels of certain genes between the two phenotype hosts of *G. fascicularis.* Under heat stress of 32 °C, the gene encoding green fluorescent protein (GFP)-like and chromosome-associated proteins, as well as genes related to oxidative phosphorylation, cell growth and death showed lower transcriptional levels in the brown *G. fascicularis* compared to the green *G. fascicularis.* Overall, the results demonstrate that the green form of *G. fascicularis* is better able to tolerate ocean warming and defend against pathogenic bacteria, likely due to higher gene transcription levels and defense ability.

**Supplementary Information:**

The online version contains supplementary material available at 10.1007/s42995-023-00190-1.

## Introduction

Coral reefs are built by reef-building corals in nutrient-depleted marine waters where they are among the most diverse and important marine ecosystems in the world (Blackall et al. 2015; Bollati et al. 2020). Reef-building corals form mutualistic associations with endosymbiotic photosynthetic algae in the Symbiodiniaceae family. This symbiotic relationship between coral and Symbiodiniaceae is the functional basis of coral reefs (LaJeunesse et al. 2018). However, environmental stressors, such as rising temperatures, can disrupt this symbiotic relationship, resulting in large-scale coral bleaching (Eakin et al. 2019; Hughes et al. 2019). Coral bleaching is becoming increasingly frequent and severe due to global warming and increased human activity (Skirving et al. 2019).

In recent years, numerous studies have found that both corals and their hosted Symbiodiniaceae have developed a variety of strategies to protect themselves from elevated temperature, revealing that certain coral species could potentially survive under future warmer conditions (Barott et al. 2020; Bollati et al. 2020; Buerger et al. 2020). For example, corals associated with *Durusdinium trenchii*, a heat-tolerant Symbiodiniaceae, often have high thermal tolerance (Jones et al. 2008). However, *D. trenchii* is not universally distributed in corals but is restricted to some coral species living in tropical habitats characterized by high temperatures (Gong et al. 2018; Leveque et al. 2019; Qin et al. 2019). Additionally, the coral hosts themselves exhibit varying degrees of thermal tolerance (Claar et al. 2020; Grottoli et al. 2014; Morikawa and Palumbi 2019). For example, recent studies have found that branched *Acropora* and *Pocilloporid* corals seem to be more vulnerable to increased temperature than massive types of *Porites* (i.e. *Porites lutea* and *Porites lobata*). The latter are able to tolerant high temperatures as they have thicker tissue, which acts as a shield and protects their symbiotic Symbiodiniaceae from severe light intensities (Claar et al. 2020; Ritson-Williams and Gates 2020). In some instances, bleached corals become exceptionally colorful rather than white. These colors derive from green fluorescent protein (GFP)-like pigments produced by the coral host (Bollati et al. 2020).

The coral’s fluorescence phenotypes, related to green fluorescent protein (GFP)-like proteins, are suggested to contribute to the host’s acclimatization to environmental stresses (Gittins et al. 2015; Jarett et al. 2017; Paley 2012). These GFP-like proteins, responsible for the prominent hues of green, red and purple-blue in reef-building corals (Alieva et al. 2008; Dove et al. 2001), can constitute up to 14% of the coral’s total soluble proteins (D’Angelo et al. 2012; Leutenegger et al. 2007; Oswald et al. 2007). The potential functions of these proteins in corals include the protection of photosynthesis of the endosymbiotic algae (D’Angelo et al. 2012; Kahng and Salih 2005), linking coral’s immune response (Palmer et al. 2009) and enabling coral-Symbiodiniaceae symbiosis (Aihara et al. 2019). The expression of GFP-like proteins in many corals is regulated by light intensity, particularly that of blue light (D’Angelo et al. 2012). Temperature is another factor affecting the synthesis of these GFP-like proteins and elevated temperatures, up to 32 °C, can significantly suppress their production (D’Angelo et al. 2008; Smith-Keune and Dove 2008). Changes to the fluorescence phenotypes of certain corals are frequently found in shallow water and during periods of high temperature, suggesting the role of fluorescent proteins (FPs) in enabling corals to acclimate to a broader range of conditions (Smith-Keune and Dove 2008; Voolstra 2020). Despite the striking incidence of fluorescence phenotypes in corals and their proposed roles, the mechanisms linking thermal bleaching tolerance in reef-building coral populations associated with fluorescence phenotypes due to GFP-like proteins remain unclear.

**Fig. 1:**
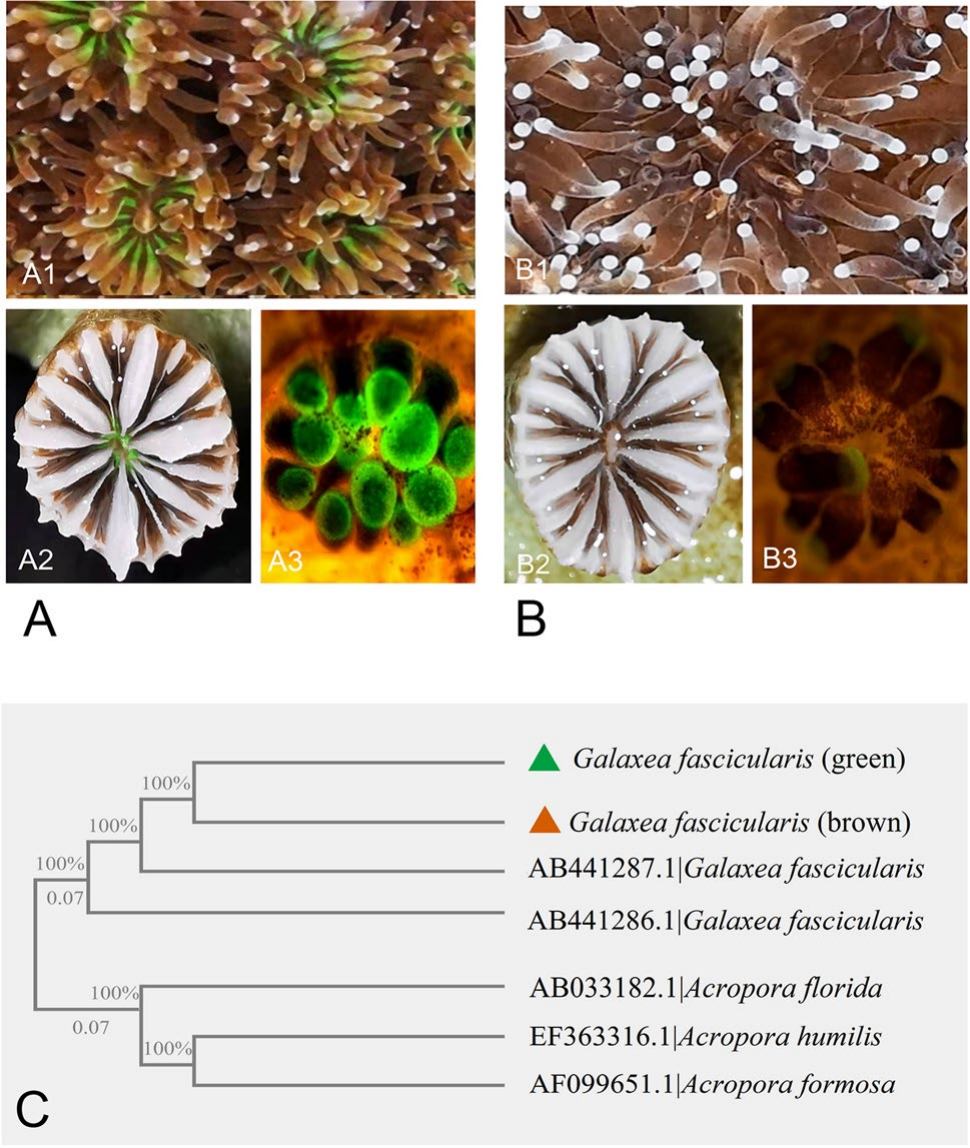
Features of the green and brown reef-building *G. fascicularis.* Representative photographs of green and brown *G. fascicularis* colonies (**A1**, **B1**). Representative photographs of corallites of green and brown *G. fascicularis* (**A2**, **B2**). Representative photographs of green and brown *G. fascicularis* under blue light excitation (**A3**, **B3**). The phylogenetic tree based on *cytb* genes was derived by the maximum likelihood method. The related *cytb* gene sequences are supplied in File S5

The reef-building coral, *Galaxea fascicularis,* is widely distributed in the Indo-Pacific Ocean (Baird et al. 2009; Ben-Zvi et al. 2015; Niu et al. 2016). In the tropical coral reef of Sanya, Hainan Island, China (109° 29′ E, 18° 12′ N), two fluorescence phenotypes (brown and green) of *G. fascicularis* (Fig. 1) were found*.* The brown *G. fascicularis* reflects the fluorescence color of Symbiodiniaceae, while the green *G. fascicularis* exhibits strong green fluorescence in its tentacles and septal areas*.* These two phenotypes are mainly associated with the thermotolerant Symbiodiniaceae species, *Durusdinium trenchii* (the relative abundance of *D. trenchii* (D1–D2–D4) > 99%, as shown in the results of this study), making them ideal for examining the role of the host’s fluorescence phenotypes in the symbiosis and acclimation processes of corals under thermal stress. Similar fluorescence phenotypes of *G. fascicularis* have also been observed in Okinawa Island, Japan (Abe et al. 2008). In this study, the physiological traits (Symbiodiniaceae density, photosynthetic performance and possible bleaching), microbiome (bacteria and Symbiodiniaceae) and metatranscriptome of the two phenotypes of *G. fascicularis* were examined in the control (29 °C) and heat stress (32 °C) treatments during a 14-day cultivation period. The aim was to identify the possible mechanisms underlying their thermal tolerance and acclimatization.

## Materials and methods

### Sample collection and experimental setup

On December 12, 2020, twelve *G. fascicularis* colonies (brown = 6, green = 6, approximately 10 cm in diameter) were collected from a tropical coral reef (at a depth of 3 m during low tide) at Sanya, Hainan Island, China (109° 29′ E, 18° 12′ N). The collected corals were transferred and acclimatized (about 14 days) in outdoor tanks (150-L). The outdoor tanks were partially shaded and exposed to natural sunlight, and received seawater pumped from Luhuitou Reef at 3 m depth. Seawater temperature in the outdoor tanks was 29 ± 0.5 °C.

**Fig. 2:**
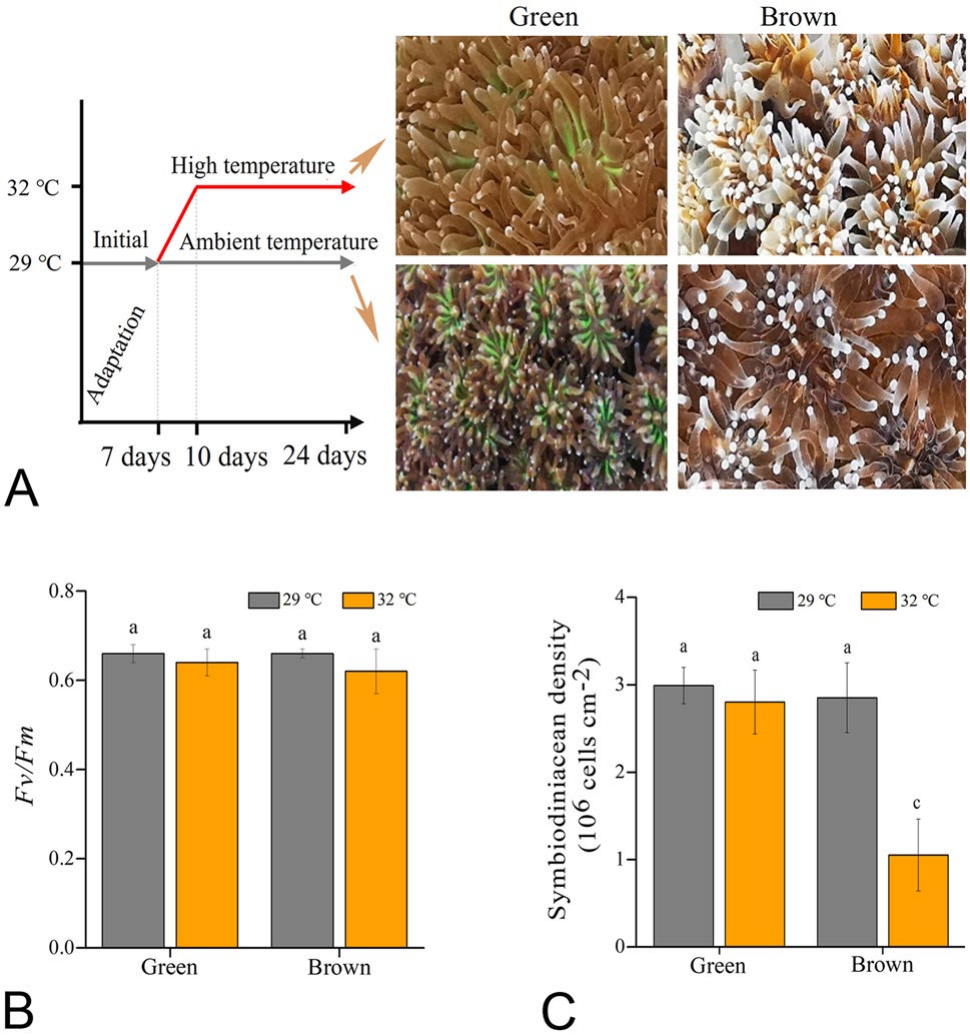
Variations in phenotypes, *F*_v_*/F*_m_ values and Symbiodiniaceae cell densities between green and brown *G. fascicularis*. Shematic depiction of the experimental design (left) and representative photographs of green and brown *G. fascicularis* under ambient and elevated temperature conditions (right) (**A**). Variations in the photochemical efficiency of PSII (*F*_v_*/F*_m_) in green and brown *G. fascicularis* under ambient and elevated temperature conditions (**B**). Variations in Symbiodiniaceae cell densities in green and brown *G. fascicularis* under ambient and elevated temperature conditions (**C**). Error bars represent the means ± SDs. One-way analysis of variance (ANOVA) was performed (*P* < 0.05, same letters: no significant difference; different letters: significant difference)

Subsequently, the coral colonies were divided into smaller branches using pincers and then acclimatized in indoor tanks (20-L) with a light intensity of 300 μmol photons m^−2^ s^−1^ (mean daytime irradiation) and a light:dark cycle of 12:12 h under 29 ± 0.5 °C. The light was provided by T5 fluorescent lamps (Giesemann, Germany) and the cultivation temperature was controlled by digital temperature controllers connected to titanium heaters (Weipro, China). Each tank contained at least 6 coral branches. After 7 days of indoor acclimation, six tanks (three for brown *G. fascicularis* and the other three for green *G. fascicularis*) were maintained at 29 ± 0.5 °C with a light intensity of 300 μmol photons m^−2^ s^−1^ and the other six tanks (three for brown *G. fascicularis* and the other three for green *G. fascicularis*) were maintained at 32 °C ± 0.5 °C with a light intensity of 300 μmol photons m^−2^ s^−1^. The temperature of 29 °C represented the mean summer ambient temperature on the reefs, while the temperature of 32 °C was about 3 °C above the bleaching threshold of local coral communities (Li et al. 2012). For the heat stress treatment, the temperature was gradually increased from 29 to 32 °C over 3 days (Fig. 2). All tanks were filled with freshly collected seawater from Sanya (in situ pH = 8.15 ± 0.003; salinity = 33.50 ± 0.015; temperature = 29.00 ± 0.006 °C; NH_4_^+^ = 10 ± 2 μg/L; NO_3_^−^ = 32 ± 2 μg/L; PO_4_^3−^ = 9 ± 1 μg/L) with a daily pre-warmed seawater renewal rate of 25%. Temperature, salinity, and pH were monitored with a pH meter (SevenGo meter, Mettler Toledo, Switzerland). Dissolved nutrients (nitrite-nitrate-NO_3_^−^, ammonia-NH_4_^+^, and phosphate-PO_4_^3−^) were analyzed using a Lachat QC8500 Flow Injection Autoanalyzer (Lachat Instruments, United States) (Supplementary Tables S1 and S2).

During the cultivations, the images of the coral colonies were taken with a GoPro (HERO9 Black, USA) for monitoring visible coral bleaching. The possible fluorescence of the corals was recorded using a fluorescence microscope under blue light excitation (Leica DMRXA, Germany). The corallites of the coral were recorded by a camera under macro mode (Canon, EOS 70D, Japan) after removing coral tissues using a Waterpik containing filtered seawater (0.45 μm).

### Symbiodiniaceae cell density and maximal PSII quantum yield (*F*_V/_*F*_M_)

Coral tissue was removed using a Waterpik containing filtered seawater (0.45 μm). The initial volume of the resulting slurry was measured with a graduated cylinder. The slurry was homogenized by votex and subsampled into four 3-mL aliquots. Subsamples were centrifuged (6500 r/min) for 5 min. After discarding the supernatant, the pellet containing algal cells were preserved in 1 mL of 5% formaldehyde at 4 °C for further analysis. The cell densities of Symbiodiniaceae were counted using replicate (*n* = 6) hemocytometer counts under microscopy (CX21, Olympus, Japan). The densities of Symbiodiniaceae cells were normalized to the coral surface area by a correlation between the weight and surface area of aluminium foil imprints (Xu et al. 2017).

The maximal photochemical quantum yield (*F*_V_*/F*_M_) of photosystem II (PS II) of the coral colonies was measured with pulse-amplitude modulation fluorometry (Diving-PAM-II, Walz, Germany). For this, the initial fluorescence (*F*_O_) of dark-adapted coral colonies (30 min) was measured under a weak modulated measuring light, and the maximum fluorescence (*F*_M_) was measured under a saturating light pulse (710 nm, 800 ms). The *F*_V_/*F*_M_ value was calculated (Hoogenboom et al. 2012) as follows:

$$\frac{{F}_{\mathrm{V}}}{{F}_{\mathrm{M}}}=\frac{{F}_{\mathrm{M}}-{F}_{\mathrm{O}}}{{F}_{\mathrm{M}}}.$$

### DNA extraction, amplification, pyrosequencing and data processing

The total genomic DNA of the coral fragments (approximately 1–2 cm^2^) was extracted using a Qiagen DNeasy Kit (Qiagen, Hilden, Germany) according to the manufacturer’s protocol. To identify Symbiodiniaceae and bacterial community compositions in corals, the ITS2 region of the Symbiodiniaceae ribosomal RNA gene and the V3 and V4 hypervariable regions of the bacterial 16S rRNA gene were PCR amplified with the ITS2 primers of ITSintfor2 (5′-GAATTGCAGAACTCCGTG-3′) and ITS2-reverse (5′GGGATCCATATGCTTAAGTTCAGCGGGT-3′), and 16S rRNA primers 341F (5′-CCTAYGGGRBGCASCAG-3′) and 806R (5′-GGACTACNNGGGTATCTAAT-3′), respectively (Gong et al. 2018, 2020a).

After pooling multiple samples in one run of Illumina sequencing (MiSeq), a unique 12-mer tag for each DNA sample was added to the 5′ ends of the primers. Each sample was amplified by PCR in a 50 μL reaction, which contained 25 μL of Multiplex Taq (Qiagen, Hilden, Germany), 10 mmol/L each primer, 60 ng of genomic DNA, and DNase-free water to a total volume of 50 μL. The cycling conditions were set as follows: 94 °C for 5 min followed by 30 cycles of denaturation at 94 °C for 30 s, annealing at 52 °C for 30 s, extension at 72 °C for 30 s, and a final extension at 72 °C for 10 min. The PCR products were validated by an Agilent 2100 Bioanalyzer (Agilent Technologies, Palo Alto, CA, USA), and quantified by a Qubit^®^ 3.0 Fluorometer (Life Technologies, New York, NY, USA). Finally, the PCR products were sequenced using a 2 × 300 paired-end (PE) configuration. Base calling was performed by the MiSeq Control Software (MCS) embedded in the Illumina MiSeq instrument.

Raw reads of the ITS2 region of the ribosomal RNA gene of Symbiodiniaceae were analysed with default settings using the SymPortal (a novel analytical framework and platform for coral-algal symbiont next‐generation sequencing-based ITS2 profiling) (Hume et al. 2019), and the 16S rRNA gene sequences were processed using the Quantitative Insights Into Microbial Ecology (QIIME1) platform as described in our previous study (Gong et al. 2020a). Statistical Analysis, using Metagenomic Profiles software (STAMP) (Parks et al. 2014), was undertaken to identify significant differences between bacterial communities associated with green and brown *G. fascicularis* under elevated temperature.

The two corals were identified based on the morphology, corallite and a molecular barcode analysis of the *cytb* (cytochrome b) gene. The *cytb* gene was amplified by PCR with previously reported primers for corals (Fukami et al. 2004) and was cloned into the pEASY’T5 Zero Cloning Vector (Transgene Biotech, Beijing, China). Clones for each coral sample were selected for further Sanger sequencing. Phylogenetic trees were constructed using the maximum likelihood (ML) method. For ML, we performed 1000 replicates of the rapid bootstrapping algorithm using MEGA-X version 10.1.8 with the Kimura 2-parameter model (Kumar et al. 2018).

### RNA extraction, sequencing and metatranscriptomic analysis

The total RNA of coral fragments (approximately 1–2 cm^2^) preserved in RNAhold^®^ (TRAN, China) was extracted using a Qiagen RNeasy Kit (Qiagen, Hilden, Germany) according to the manufacturer’s protocol. RNA quantity and integrity were analysed using a NanoDrop ND-1000 spectrometer (Wilmington, DE, USA) and an Agilent 2100 Bioanalyzer (Santa Clara, CA, USA). RNA samples with high purity (OD260/280 between 1.9 and 2.1) and high integrity [RNA integrity number (RIN) > 8.5] were used for further cDNA library construction. cDNA library construction and the sequencing and quality control of raw sequences were performed according to our previous study (Gong et al. 2020b).

The quality control and analysis of raw reads were performed using SqueezeMeta software, a fully automatic pipeline for metagenomic/metatranscriptomic analysis (Tamames and Puente-Sánchez 2019). In brief, Trimmomatic-0.38 software was used for adaptor removal, trimming and filtering by quality according to default parameters (Bolger et al. 2014). The obtained clean reads were further assembled using Megahit (Li et al. 2015). Diamond software (Buchfink et al. 2015) was used for homology searching of assembled gene sequences against several taxonomic and functional databases, including the eggNOG database (Huerta-Cepas et al. 2016), the latest publicly available version of the KEGG database (Ogata et al. 1999) and the PFAM database using HMMER3 (Finn et al. 2014) with default settings. For taxonomic assignment, an LCA algorithm that searches for the last common ancestor of the hits for each query assembled gene using the results of the Diamond search against the GenBank nr database (the annotated genomes of *Acropora*, *Pocillopora* and Symbiodiniaceae (*Symbiodinium microadriacticum*, *Breviolum minutum*, *Cladocopium goreaui*, *D. trenchii*, *Fugacium kawagutii*) were included in this database) was applied (Buitrago-López et al. 2020; González-Pech et al. 2021; Shinzato et al. 2021; Tamames and Puente-Sánchez 2019). To estimate the abundance of each assembled gene in each sample, original reads were mapped onto the contigs resulting from the assembly using Bowtie2 software (Langmead and Salzberg 2012). RSEM software (Li and Dewey 2011) was used to compute the average coverage and normalized TPM values that provide information on gene abundance.

The analysis of differentially transcribed genes among different samples was performed using the DESeq2 method, with a threshold *P* value of ≤ 0.05, fold change ≥ 2 (Liu et al. 2017; Love et al. 2014).

### Statistical analysis

The physiological data (Symbiodiniaceae density, photosynthetic performance) are presented as the mean ± standard deviation of six independent biological replicates. The microbiome data and metatranscriptome data are presented as three independent biological replicates. One-way analysis of variance (ANOVA) with Tukey’s test was applied to analyse the physiological and gene transcriptional data with a confidence level of 0.05. All analyses and visualizations of the results were performed with the vegan and heatmap packages in R software (R 3.1.2) and/or Origin 8.5 software.

## Results

### Morphological and physiological traits and responses to elevated temperature

**Fig. 3:**
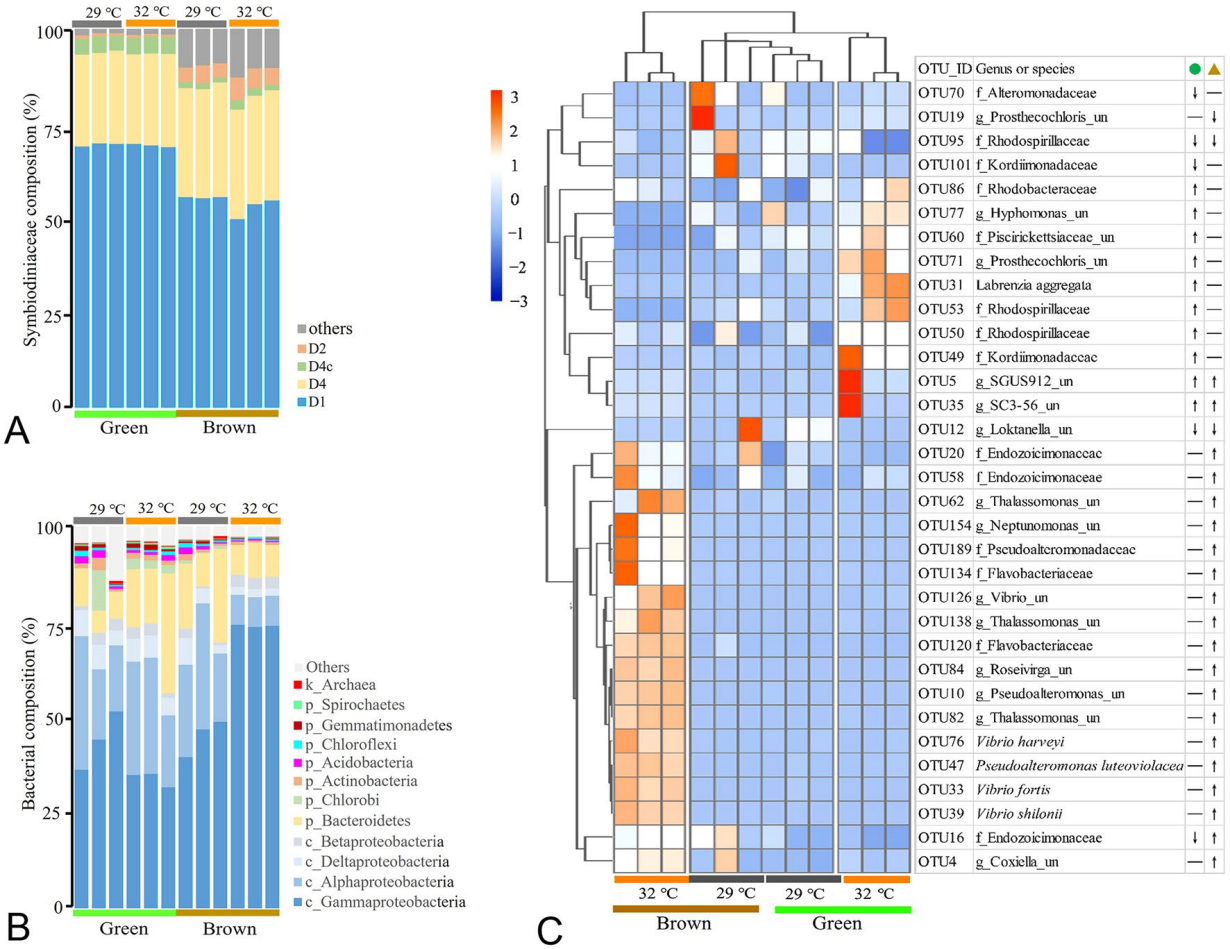
Variations in Symbiodiniaceae and bacterial composition in green and brown *G. fascicularis*. Symbiodiniaceae composition in green and brown *G. fascicularis* under ambient and elevated temperature conditions (**A**). Bacterial composition in green and brown *G. fascicularis* under ambient and elevated temperature conditions (**B**). Pheatmap of significantly changed bactierial groups (with total abundance over 50) associated with green and brown *G. fascicularis* under high temperature (**C**)

The morphological and physiological traits of brown and green *G. fascicularis* under control and heat stress treatments are shown in Figs. 1, 2 and 3. The colonies (Fig. 1A1, B1) and corallities (Fig. 1A2–A3, B2–B3) of the two phenotypes showed discernible differences in their color morphology. Notably, the tentacles and septa of the *G. fascicularis* with the green phenotype displayed a stronger green fluorescence compared to that of brown *G. fascicularis* when exposed to blue light (Fig. 1A3). The phylogenetic analysis, based on *cytb* gene, revealed that both phenotypes of *G. fascicularis* were grouped together with that of *G. fascicularis* (AB441286.1 and AB441286.1) from Okinawa, Japan, with 100% sequences similarity and bootstrap values (Fig. 1C)*.* The control treatment (29 °C) did not result in bleaching in either phenotype of *G. fascicularis* (Fig. 2A, Supplementary Fig. S1) and *Fv*/*Fm* (Fig. 2B) and Symbiodiniaceae cell density (Fig. 2C) remained consistent. However, in the heat stress treatment (32 °C), brown *G. fascicularis* exhibited noticeable bleaching (Fig. 2A, Supplementary Fig. S1), accompanied by a reduction in Symbiodiniaceae cell density from 2.9 × 10^6^ to 1.1 × 10^6^ cells cm^−2^ (Fig. 2C). No such changes were observed in the green *G. fascicularis* (Fig. 2)*.*

### Microbiome of the explored corals and its responses to elevated temperature

In the control treatment the two phenotypes primarily hosted *D. trenchii* (D1–D2–D4c, with relative abundances > 99%) and *Cladocopium* sp. (C3–C21, with relative abundances < 1%) of Symbiodiniaceae (Fig. 3A). Proteobacteria, Betaproteobacteria, Bacteroidetes, Chlorobi, Actinobacteria, Chloroflexi, Gemmatimonadetes and Spirochaetes were the dominant bacteria associated with both phenotypes (Fig. 3B)*.* At an elevated temperature of 32 °C, the composition of the Symbiodiniaceae did not undergo notable change (Fig. 3A) but there was a notable effect on the bacterial community composition of the two phenotypes (Fig. 3B, C). At the phylum level, the relative abundance of Proteobacteria increased from approximately 50 to 75% in brown *G. fascicularis* (Fig. 3B, *P* value of < 0.05) and that of Bacteroidetes increased from approximately 7 to 20% in green *G. fascicularis* (Fig. 3B, *P* value < 0.05)*.* At a fine-scale taxonomic level (Fig. 3C and Supplementary Fig. S5)*,* the relative abundances of two OTUs affiliated with Amoebophilaceae, which are potential endosymbiotic bacteria, also increased over twofold in both brown and green *G. fascicularis* under elevated temperature (*P* value < 0.05). Notably, the relative abundances of OTUs affiliated with *Labrenzia aggregata* and OTUs affiliated with *Prosthecochloris* increased over 70-fold in green *G. fascicularis* under elevated temperature (*P* value < 0.05). In bleached brown *G. fascicularis*, the relative abundances of OTUs affiliated with pathogenic bacteria*,* i.e., *Vibrio shilonii*,* Vibrio harveyi*,* Vibrio fortis*, *Coxiella* and *Thalassomonas*, increased over tenfold (*P* value < 0.05).

### Metatranscriptomic profiles of the corals and their response to elevated temperature

**Fig. 4:**
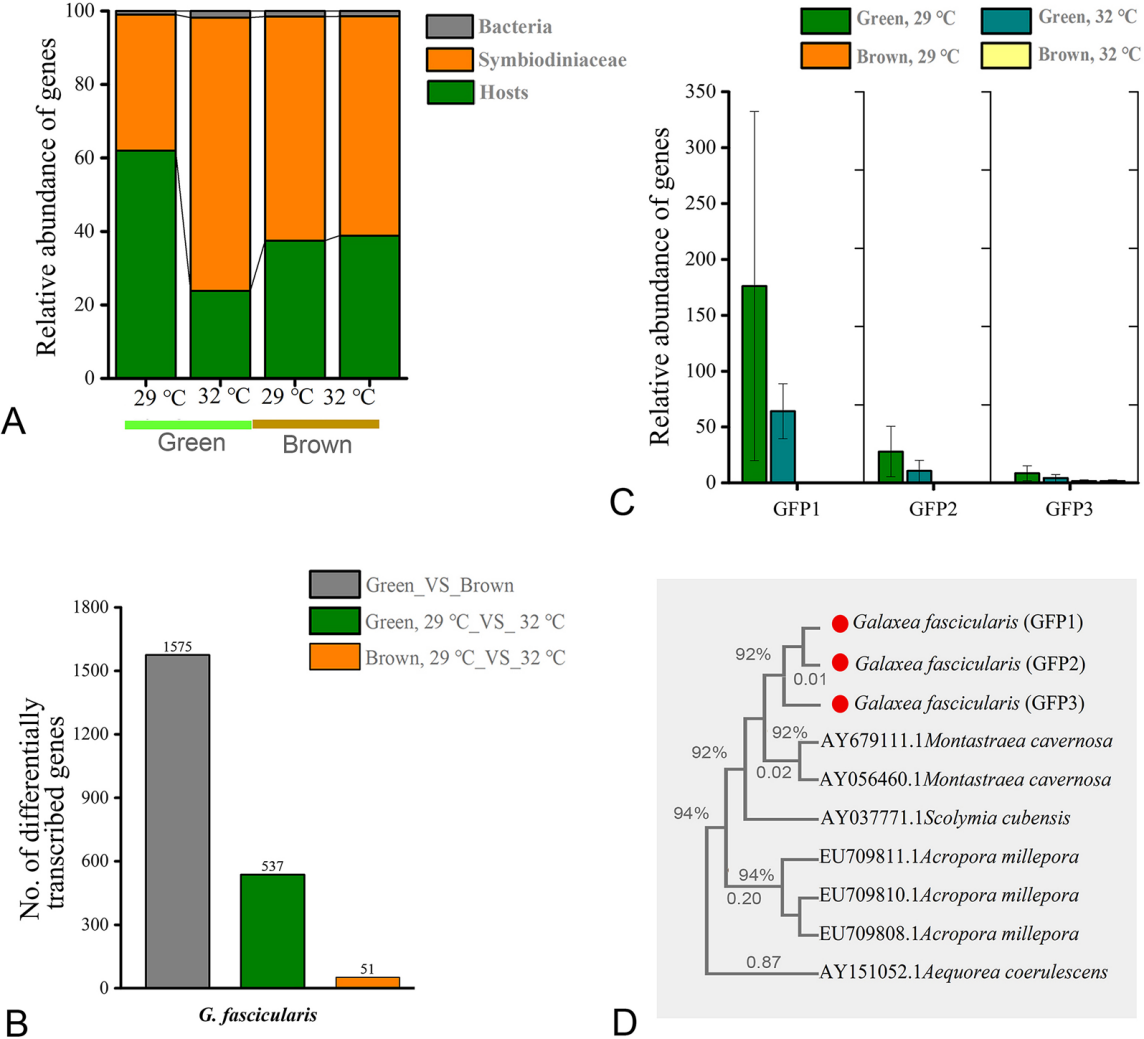
Metatranscriptomic profiles of green and brown *G. fascicularis*. Total abundance of actively transcribed genes belonging to coral hosts, symbionts (Symbiodiniaceae) and bacteria (**A**). Number of differentially transcribed genes in the *G. fascicularis* host under different conditions (**B**)*.* Relative abundance of transcripts encoding GFP-like protein in green and brown *G. fascicularis* under different conditions (**C**). The phylogenetic tree based on sequences of transcripts encoding GFP was derived by the maximum likelihood method (**D**). Error bars represent the means ± SDs. One-way analysis of variance (ANOVA) was performed (*P* < 0.05, same letters: no significant difference; different letters: significant difference)

The metatranscriptomic profiles of the two *G. fascicularis* phenotypes showed that more than 98% of total reads were affiliated with the coral hosts (ranging from 23 to 61%) and Symbiodiniaceae (ranging from 37 to 74%), with bacterial reads making up less than 2% (Fig. 4A). The genes that were significantly transcribed (with a *P* value of < 0.05 and a fold change of ≥ 2) in different groups or under varying temperatures were found to be affiliated primarily with the coral hosts themselves (Fig. 4B).

**Fig. 5:**
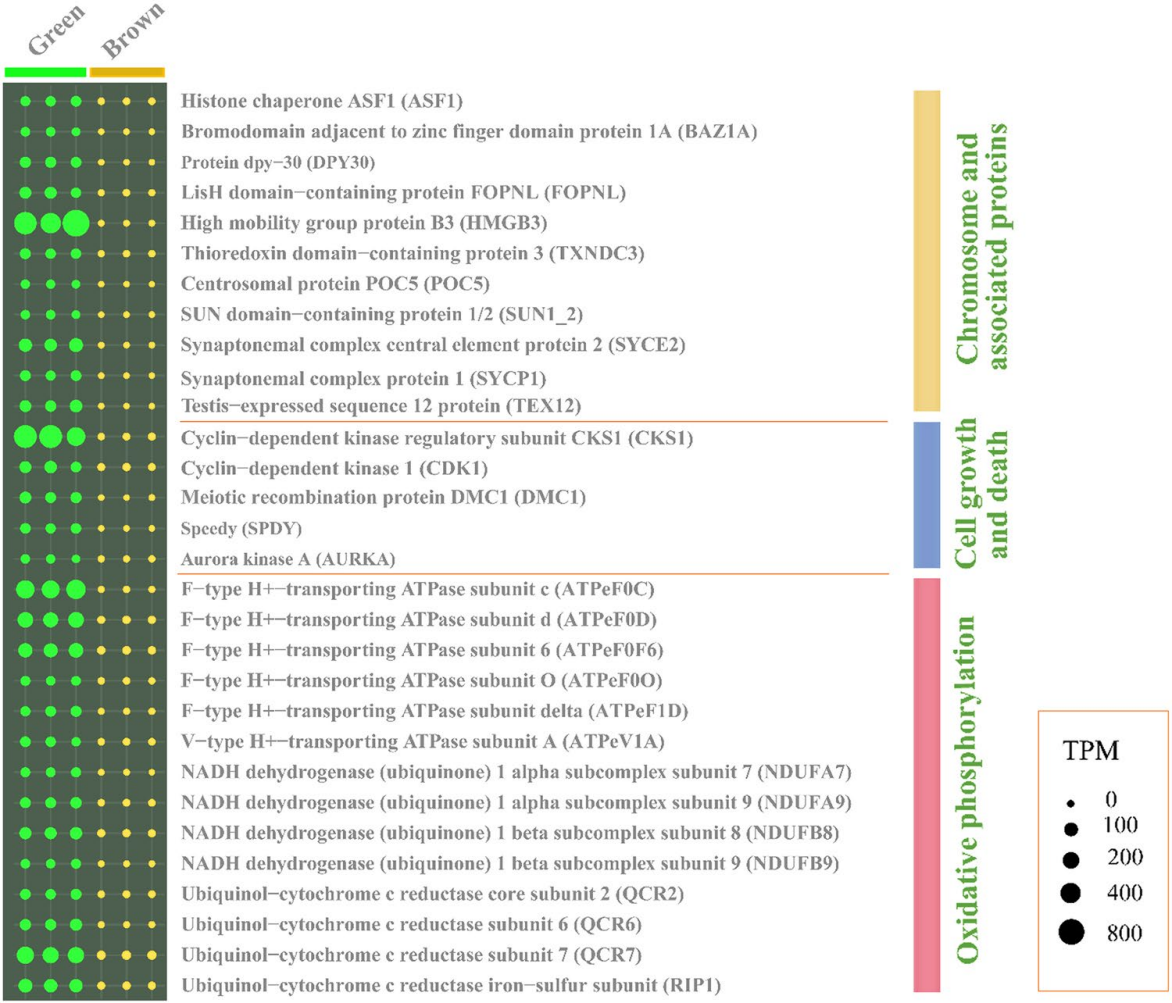
Bubble chart of based on the relative abundance of differentially transcribed genes (*P* < 0.05, total TMP > 50) between green and brown *G. fascicularis*

The transcriptomic profiles of the green and brown *G. fascicularis* at 29 °C were compared and it was found that 1575 genes were differentially transcribed between the two phenotypes (Fig. 4B). Notably, the gene (transcript) encoding green fluorescent-like protein (GFP) was highly transcribed in green *G. fascicularis*, with one transcript showing a more than 400-fold increase (designated as GFP1, *P* value of < 0.05, Fig. 4C). Phylogenetic analysis revealed that the nucleotide sequences of the three GFP transcripts of *G. fascicularis* were clustered with those of other reef-building corals and *Aequorea coerulescens* (jellyfish, Fig. 4D, Supplementary Files S1–S2), indicating homology. Other highly transcribed genes in the green *G. fascicularis* were related to oxidative phosphorylation, cell growth and death, chromosome and associated proteins (Fig. 5). Specifically, the transcription levels of genes encoding ubiquinol-cytochrome c reductase subunit 7 (QCR7), F-type H+-transporting ATPase subunit c (ATPeF0C), cyclin-dependent kinase regulatory subunit CKS1 (CKS1) and high mobility group protein B3 (HMGB3) increased tenfold (*P* value of < 0.05) higher in green *G. fascicularis* compared to brown *G. fascicularis* (Fig. 5 and Supplementary File S3).

**Fig. 6:**
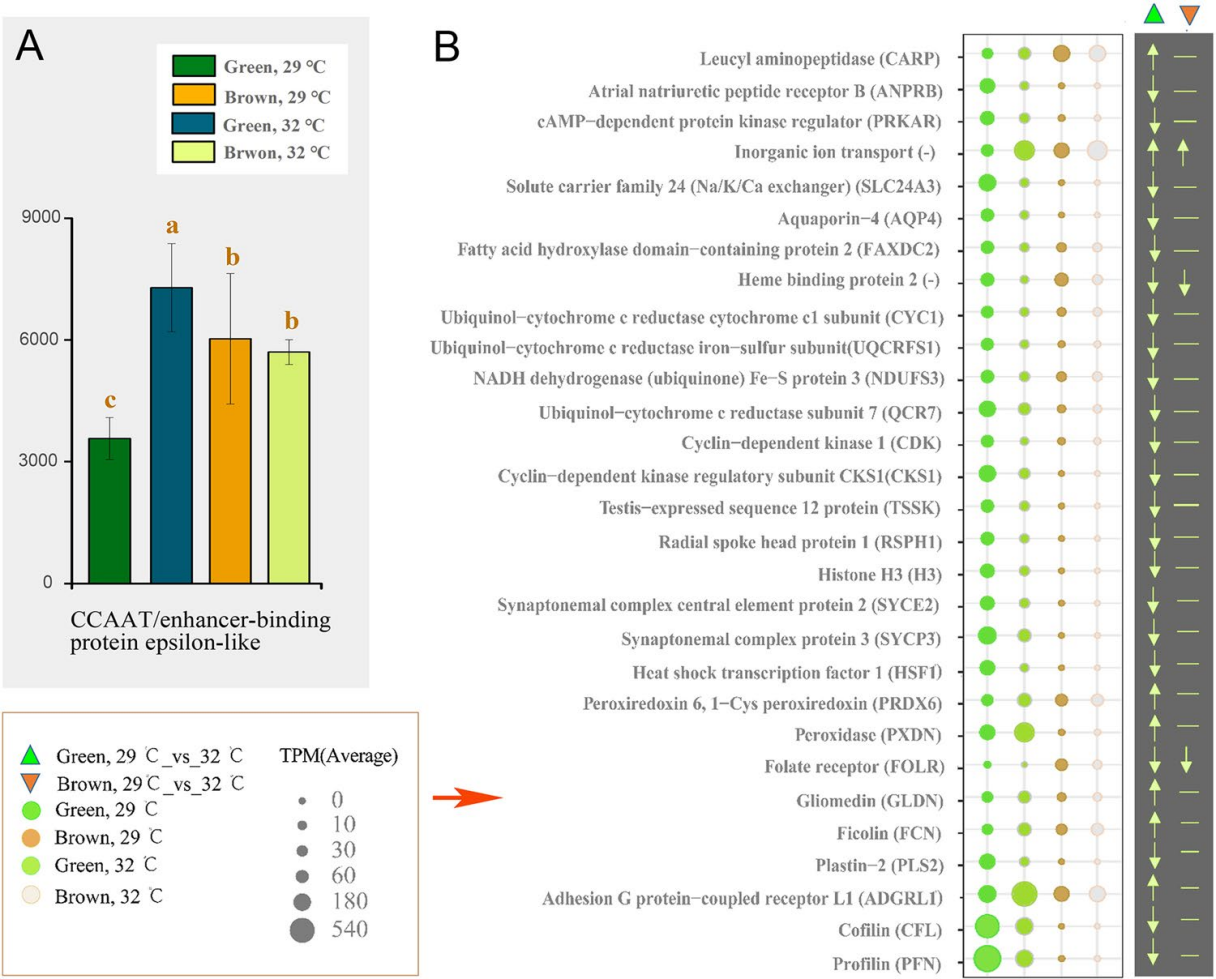
Differentially transcribed genes of green and brown *G. fascicularis* hosts under elevated temperature. The relative abundance of the gene encoding CCAAT/enhancer-binding protein epsilon-like (Cebpe, **A**) in green and brown *G. fascicularis* under elevated temperature. Bubble chart of differentially transcribed genes (*P* < 0.05, total TMP > 50) between green and brown *G. fascicularis* under under elevated temperature (**B**). Error bars represent the means ± SDs. One-way analysis of variance (ANOVA) was performed (*P* < 0.05, same letters: no significant difference; different letters: significant difference)

To further explore the molecular basis of the underlying differences in thermal tolerance between the two phenotypes, a comparison was made of the transcriptomic profiles at 29 °C and 32 °C. A total of 537 and 51 genes were differentially transcribed in green *G. fascicularis* (29 °C vs 32 °C) and brown *G. fascicularis* (29 °C vs 32 °C) groups, respectively (Fig. 4B). It was observed that the most abundant gene encoding CCAAT/enhancer-binding protein epsilon-like (Cebpe), possibly involved in the host’s defense response to pathogenic bacteria, was significantly upregulated in green *G. fascicularis* (Fig. 6A, *P* value of < 0.05)*.* Moreover, the genes encoding profilin (PFN), cofilin (CFL), adhesion G protein-coupled receptor L1 (ADGRL1), peroxidase (PXDN), ubiquinol-cytochrome c reductase subunit 7 (QCR7), cyclin-dependent kinase regulatory subunit CKS1 (CKS1), which are involved in cell–cell interactions and adhesion, antioxidant activity, oxidative phosphorylation, and the cell cycle, were differentially transcribed in the two corals under elevated temperature (*P* value of < 0.05) (Fig. 6B and Supplementary File S4).

## Discussion

Corals are known to produce large amounts of FPs, homologous to the popular GFP, which are responsible for the conspicuous green, red and purple-blue colouration of reef-building corals (Gittins et al. 2015). These proteins are believed to have photoprotective properties and are thus considered mechanisms of a coral’s acclimatization to environmental stresses (Gittins et al. 2015; Jarett et al. 2017; Paley 2012). In this study, it was observed that green *G. fascicularis* had higher green fluorescence intensity and greater transcription of the gene encoding GFP-like proteins than brown *G. fascicularis*. The gene encoding the GFP-like proteins of *G. fascicularis* was homologous to the typical GFP from that of *A. coerulescens* (jellyfish) (Gurskaya et al. 2001). Similarly, a previous study on *Acropora tenuis* found that yellow-green *A. tenuis* expressed higher level of green fluorescence than brown or purple *A. tenuis* (Satoh et al. 2020). Those findings suggest that differences in gene transcription of FPs may underlie the diverse fluorescent phenotypes of coral.

Fluorescence phenotypes were further linked to the thermal bleaching tolerance of coral hosts. After 14 days of cultivation with a temperature increase of 3 °C, notable bleaching occurred in brown *G. fascicularis* but not in the green. This indicates that the green phenotype is thermally resistant and brown *G. fascicularis* is not. Thus it is proposed that if this feature is heritable, *G. fascicularis* may have adapted to thermal stress by developing new phenotypes related to the expression of FPs. A similar study in Okinawa found that reef-building *A. tenuis* with the green fluorescence phenotypes has greater potential resistance to increased temperatures in the summer (Satoh et al. 2020).

The ability of coral to withstand thermal bleaching is complex, varying across colonies, taxa, and environments (Barott et al. 2020; Morikawa and Palumbi 2019). Reef-building corals form obligate endosymbioses with photosynthetic Symbiodiniaceae, and different Symbiodiniaceae associated with coral hosts contribute to the differential bleaching susceptibility (Botana 2019; Bollati et al. 2020; Claar et al. 2020). For example, *D. trenchii* is generally more heat-resistant than *Cladocopium* sp. (Buerger et al. 2020; Karim et al. 2015; Osman et al. 2018) and the coral hosting *D. trenchii* tend to exhibit higher thermal tolerance (Buerger et al. 2020). Photoinhibition of Symbiodiniaceae cells, caused by elevated temperatures, can lead to coral bleaching through the production of reactive oxygen species (ROS diffuse into the host tissue (Douglas 2003). Previous studies have suggested that the GFP-like proteins in corals might play roles in the protection of photosynthesis of endosymbiotic algae, enhancing the thermal tolerance of coral hosts (D’Angelo et al. 2012; Kahng and Salih 2005). However, this study revealed that the Symbiodiniaceae hosted by green and brown *G. fascicularis* maintained similar photosynthetic activity and gene transcription profiles under both ambient and elevated temperatures. The causes of this might be that these two phenotypes are mainly associated with the thermotolerant *D. trenchii* and the heat stress of 32 °C is within a suitable growth temperature range of this endosymbiotic algal genus (Iglesias-Prieto et al. 1992; Karim et al. 2015; LaJeunesse et al. 2018; Roberty et al. 2016). Therefore, the results presented here do not support the previously proposed roles of fluorescent proteins in corals for light protection of the endosymbiotic Symbiodiniaceae avoiding bleaching caused by ROS production (D’Angelo et al. 2012; Kahng and Salih 2005).

Interestingly, the current results reveal that the varying thermal bleaching tolerance of the two *G. fascicularis* with different phenotypes was linked to differential gene transcription, related to GFP-like proteins and the core metabolism of the hosts. It was observed that green *G. fascicularis* displayed a different gene transcription profile from that of brown *G. fascicularis.* The green *G. fascicularis* exhibited a high transcription level of the gene encoding GFP at both ambient and elevated temperatures. Given its bleaching tolerance, it was anticipated that the heat stress resistance of the green *G. fascicularis* would be influenced by the expression levels of the GFP gene. Additionally, high transcription of genes involved in core metabolism (i.e., respiration, the cell cycle and growth) was also noted in the green *G. fascicularis.* Hence, it was expected that different control mechanisms of GFP gene transcription, along with host metabolitic function-associated genes, would elucidate the variability in bleaching tolerance.

**Fig. 7:**
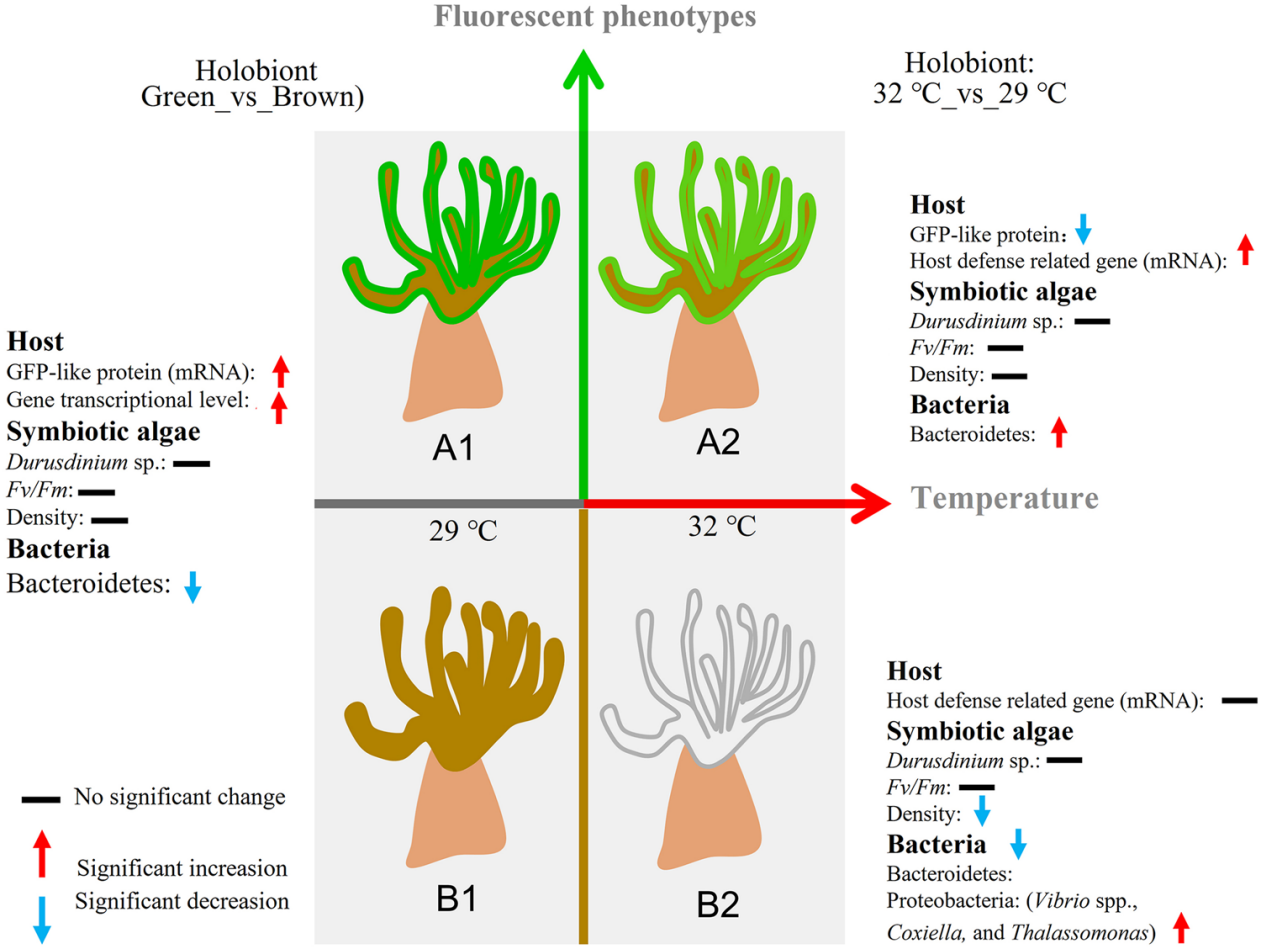
A proposed model of fluorescence phenotypes-linked thermal bleaching tolerance in *G. fascicularis.* **A1** Green *G. fascicularis* under ambient temperature. **A2** Green *G. fascicularis* under elevated temperature. **B1** Brown *G. fascicularis* under ambient temperature. **B2** Brown *G. fascicularis* under elevated temperature

This study found that several pathogenic bacteria*,* including *V. shilonii*,* V. harveyi*,* V. fortis*, *Coxiella* and *Thalassomonas*, were enriched more than 10 times in bleached brown *G. fascicularis* under elevated temperatures (*P* value of < 0.05). In fact, *V. shilonii*,* V. harveyi*,* V. fortis* and *Thalassomonas* have been reported as pathogenic bacteria that directly bleach or lyse the endosymbiotic Symbiodiniaceae hosted by corals (Luna et al. 2009; Thompson et al. 1986), although the exact mechanism remains unknown. *Coxiella* (*Coxiella burnetii*) is an obligate intracellular bacterium, responsible for Q fever, that survives in mammalian macrophages (Benoit et al. 2008). The present study is the first to report the association of *Coxiella* with coral thermal bleaching, as the relative abundance of this genus was significantly increased in bleached coral. It would be interesting to examine whether *Coxiella* species, as possible pathogenic bacteria of coral, are responsible for coral bleaching. Taken together, the results indicate that the bleaching of brown *G. fascicularis* was probably caused by these pathogenic bacteria under high temperature (Fig. 7).

DMSP (dimethylsulfoniopropionate) and DMS (dimethylsulfide) are important compounds in the global sulfur cycle. Previous studies have shown that the DMSP in coral holobionts is produced by both coral and Symbiodiniaceae (Raina et al. 2013). The bacteria detected in corals, such as *L. aggregate*, might be able to degrade the generated DMSP into the climate-active gas DMS via the bacterial cleavage pathway (Zhong et al. 2021). *Prosthecochloris* (photoautotrophic bacteria) participates in sulfur metabolism. Hydrogen sulfide is toxic to a wide range of eukaryotic organisms and can lead to the initiation of coral blank band disease. *Prosthecochloris*, serving as potential sulfur-oxidizing bacteria, might oxidize holobiont-accumulated hydrogen sulfide to sulfate, thus contributing to coral health through detoxification of reduced sulfur compounds (Gong et al. 2020a). Therefore, the changes in the abundance of *L. aggregata* and *Prosthecochloris* might be important for regulating nutrient metabolism in coral holobiont under elevated temperature.

## Conclusions

In this study, the impact of elevated temperature on two phenotypes of *G. fascicularis* was examined. Results indicate that the physiology, composition, and gene function of the Symbiodiniaceae associated with both green and brown *G. fascicularis* were not significantly affected by the elevated temperature. This is at variance with the previously proposed theory that fluorescent proteins (FPs) protect the photosynthesis of Symbiodiniaceae and prevent coral bleaching induced by ROS under increased temperatures. However, distinct differences in gene transcription levels and bleaching tolerance between the two phenotypes of *G. fascicularis* under thermal stress were observed. Green *G. fascicularis* exhibited higher transcriptional levels for gene encoding GFP and genes involved in core metabolic pathways. In comparison, brown *G. fascicularis* was more susceptible to bleaching, and showed an increased relative abundance of potentially pathogenic bacteria. In summary, this study provides important new insights into the inter-phenotype differences in the thermal bleaching of *G. fascicularis*. These differences appear to be related to host metabolic activity and defense mechanisms against pathogenic bacteria, which are positively correlated with host fluorescence phenotypes.

### Supplementary Information

Below is the link to the electronic supplementary material.

Supplementary file1 (CSV 3 KB)

Supplementary file2 (FAS 8 KB)

Supplementary file3 (CSV 5 KB)

Supplementary file4 (CSV 5 KB)

Supplementary file5 (FAS 6 KB)

Supplementary file6 (DOCX 13 KB)

Supplementary file7 (DOCX 2490 KB)

## Data Availability

The data generated as part of this study are access controlled. The raw sequence data (a total of twelve RNA sequencing libraries, twelve 16S rRNA sequencing libraries and twelve ITS2 sequencing libraries) produced in this study were deposited in the Sequence Read Archive (PRJNA763702 and PRJNA764551) of the NCBI (https://blast.ncbi.nlm.nih.gov). The source data underlying all the figures are provided as supplementary data files.
