# Supplementary material for: Linking coral fluorescence phenotypes to thermal bleaching in the reef-building Galaxea fascicularis from the northern South China Sea
Source: Mar Life Sci Technol. 2023 Oct 18;6(1):155–67. doi: 10.1007/s42995-023-00190-1 (PMC10902222; doi:10.1007/s42995-023-00190-1)
Supplement: Supplementary file 6 — Supplementary file6 (DOCX 13 KB) [file 42995_2023_190_MOESM6_ESM.docx]

**Script for bacteria 16S analysis**

$ split_libraries_fastq.py -i yourmerged.fastq(1.fastq,2.fastq,3.fastq......) -o split -m mapping.txt --barcode_type 'not-barcoded' --sample_id yourfastqid(1,2,3.....)

$ pick_otus.py -i split/seqs.fna -o otus -m uclust_ref -s 0.97 -z -r $ /usr/local/lib/python2.7/dist-packages/qiime_default_reference/gg_13_8_otus/rep_set/97_otus.fasta

$ pick_rep_set.py -i otus/seqs_otus.txt -f split/seqs.fna -o rep_otu/rep_set.fna

$ align_seqs.py -i  rep_otu/rep_set.fna -o align -m pynast -a uclust -p 0.75 -t /usr/local/lib/python2.7/distpackages/qiime_default_reference/gg_13_8_otus/rep_set_aligned/85_otus.pynast.fasta

$ ilter_alignment.py -i align/rep_set_aligned.fasta -o filter

$ make_phylogeny.py -i filter/rep_set_aligned_pfiltered.fasta -o phylogeny/rep_set.tre -t fasttree

$ assign_taxonomy.py -i rep_otu/rep_set.fna -r /usr/local/lib/python2.7/dist-packages/qiime_default_reference/gg_13_8_otus/rep_set/97_otus.fasta -t $/usr/local/lib/python2.7/dist-packages/qiime_default_reference/gg_13_8_otus/taxonomy/97_otu_taxonomy.txt -m uclust

$make_otu_table.py -i otus/seqs_otus.txt -t uclust_assigned_taxonomy/rep_set_tax_assignments.txt -o otu_table/otu_table.biom

**Script for Symbiodiniaceae ITS2 analysis**

$ ./main.py --load /path of raw fastq sequences/ --name xxx--num_proc 3

$ nohup ./main.py --load / path of data sheet / --name xxx --num_proc 3 --data_sheet

**Script for coral metatranscrptome data analysis**

$ nohup /path/SqueezeMeta.pl -m coassembly –p xxx -s ./xxx.samples -f ./ -miniden 50 &
