# Supplementary material for: Linking coral fluorescence phenotypes to thermal bleaching in the reef-building Galaxea fascicularis from the northern South China Sea
Source: Mar Life Sci Technol. 2023 Oct 18;6(1):155–67. doi: 10.1007/s42995-023-00190-1 (PMC10902222; doi:10.1007/s42995-023-00190-1)
Supplement: Supplementary file 7 — Supplementary file7 (DOCX 2490 KB) [file 42995_2023_190_MOESM7_ESM.docx]

**Supplementary Data for:**

**Linking fluorescence phenotypes to thermal bleaching in the coral** ***Galaxea fascicularis* from the northern South China Sea**

**Sanqiang Gong^1,2†^ ⋅ Jiayuan Liang^3†^ ⋅ Gang Li^1,2^ ⋅** **Lijia Xu^4^ ⋅ Yehui Tan^1,2^ ⋅ Xinqing Zheng^5^ ⋅ Xuejie Jin^1^ ⋅ Kefu Yu^3*^ ⋅ Xiaomin Xia^1,2^****^*^**

^1^Key Laboratory of Tropical Marine Bio-resources and Ecology & Guangdong Provincial Key Laboratory of Applied Marine Biology, South China Sea Institute of Oceanology, Chinese Academy of Sciences, Guangzhou 510301, China

^2^Southern Marine Science and Engineering Guangdong Laboratory (Guangzhou), Guangzhou 510301, China

^3^Coral Reef Research Center of China, Guangxi University, Nanning 53004, China

^4^South China Institute of Environmental Sciences, The Ministry of Ecology and Environment of PRC, Guangzhou 510530, China

^5^Third Institute of Oceanography, Ministry of Natural Resources, Xiamen 361005, China

^†^These authors contributed equally to this work

*****Corresponding author:

Xiaomin Xia

TEL: +86-20-31955147

E-mail: [xiaxiaomin@scsio.ac.cn](mailto:xiaxiaomin@scsio.ac.cn)

Kefu Yu

Email: kefuyu@scsio.ac.cn

**Table S1** Experimental seawater conditions for ambient temperature.

| Treatment | pH | Temperature  (℃) | Salinity | NH_4_^+^  (μg/L) | NO_3_^−^  (μg/L) | PO_4_^3−^  (μg/L) |
| --- | --- | --- | --- | --- | --- | --- |
| In situ | 8.15±0.003 | 29.00±0.006 | 33.50±0.015 | 10±2 | 32±2 | 9±1 |
| Day 0 | 8.15±0.003 | 28.90±0.006 | 33.50±0.012 | 11±2 | 33±2 | 7±1 |
| Day 7 | 8.13±0.003 | 29.10±0.008 | 33.50±0.011 | 14±3 | 34±3 | 6±1 |
| Day 14 | 8.15±0.002 | 29.04±0.004 | 33.50±0.012 | 12±1 | 32±1 | 8±2 |
| Day 24 | 8.15±0.003 | 29.10±0.006 | 33.50±0.012 | 10±1 | 32±3 | 7±3 |

**Table S2** Experimental seawater conditions for elevated temperature.

| Treatment | pH | T  (℃) | Salinity | NH_4_^+^  (μg/L) | NO_3_^−^  (μg/L) | PO_4_^3−^  (μg/L) |
| --- | --- | --- | --- | --- | --- | --- |
| In situ | 8.15±0.003 | 29.00±0.006 | 33.50±0.014 | 10±2 | 32±2 | 9±1 |
| Day 0 | 8.15±0.003 | 29.10±0.006 | 33.50±0.011 | 12±2 | 34±1 | 8±3 |
| Day 7 | 8.13±0.003 | 29.10±0.008 | 33.50±0.012 | 15±3 | 35±4 | 9±1 |
| Day 14 | 8.15±0.002 | 32.50±0.009 | 33.50±0.012 | 13±1 | 33±3 | 9±3 |
| Day 24 | 8.15±0.003 | 32.10±0.004 | 33.50±0.013 | 12±1 | 32±2 | 11±4 |

**
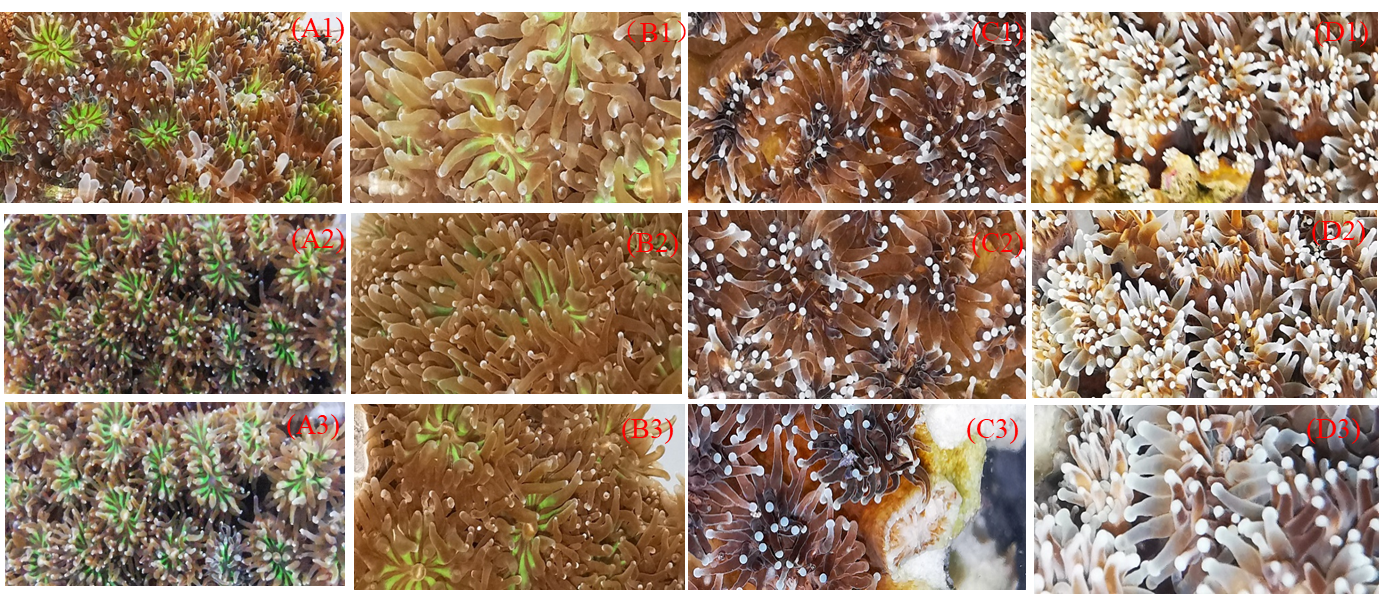
**

**Fig. S1** Representative photographs of green and brown *G. fascicularis* under ambient and elevated temperature conditions. A-B: green *G. fascicularis*. C-D: brown *G. fascicularis*. A1-A3,C1-C3: under ambient temperature. B1-B3, D1-D3: under elevated temperature.
